# Area of Wharton's jelly as an estimate of the thickness of the umbilical cord and its relationship with estimated fetal weight

**DOI:** 10.1186/1742-4755-8-32

**Published:** 2011-11-04

**Authors:** Cristiane Barbieri, Jose G Cecatti, Fernanda G Surita, Maria L Costa, Emilio F Marussi, Jose V Costa

**Affiliations:** 1 Department of Obstetrics and Gynecology, School of Medical Sciences, Universidade Estadual de Campinas - UNICAMP, Campinas, São Paulo, Brazil

**Keywords:** ultrasonography, pregnancy, umbilical cord, umbilical vessels, Wharton's jelly

## Abstract

**Background:**

To build a reference curve for the area of Wharton's jelly (WJ) in low-risk pregnancies from 13 to 40 weeks and to assess its relationship with estimated fetal weight (EFW).

**Methods:**

2,189 low-risk pregnancies had the area of WJ estimated by ultrasound and the 10^th^, 50^th ^and 90^th ^percentiles calculated using a third-degree polynomial regression procedure. EFW by ultrasound was correlated with the measurement of the area of WJ.

**Results:**

The area of WJ increased according to gestational age (R^2 ^= 0.64), stabilizing from the 32^nd ^week onwards. There was a significant linear correlation between area of WJ and EFW up to 26 weeks (R = 0.782) and after that 5t remained practically constant (R = 0.047).

**Conclusion:**

The area of WJ increases according to gestational age, with a trend to stabilize at around 32 weeks of gestation. It is also linearly correlated with EFW only up to 26 weeks of gestation.

## Background

The umbilical cord is responsible for maternal-fetal blood flow. Normally, it is composed of two arteries permeated with venous blood and a vein that transports arterial blood, cushioned by a special type of mucous connective tissue known as Wharton's jelly (WJ) and by remnants of the allantoids [1].

WJ consists of a fundamental amorphous substance containing glycosaminoglycans, proteoglycans and, predominantly, hyaluronic acid. It also contains cells with similar characteristics of smooth muscle ones and that allows its contractile function. These cells constitute an interconnected network of collagen that form canaliculi and perivascular spaces [2,3], permitting adequate blood flow to the fetus in cases of umbilical cord compression during pregnancy or delivery [4].

Alterations in the area of WJ have been described in various conditions such as hypertensive disease [5,6], tobacco smoking [6], prematurity and fetal distress during labor [7]. The absence of WJ around vessels of the umbilical cord has been found in cases of perinatal mortality [8], whereas the presence of a large area of WJ has been described in cases of diabetes mellitus [9]. Until recently, data on WJ abnormalities consisted of findings resulting from pathological examinations or case reports [10]. With recent progress achieved in ultrasonographic techniques during pregnancy, several investigators have concentrated their efforts on studying the umbilical cord and its components.

The presence of a thin cord identified during pregnancy places the fetus at risk of restricted growth and birthweight, classified as small for gestational age. This appears to be a consequence of a reduction in the area of WJ. Therefore, in 2001, a reference curve for the area of WJ in accordance with fetal biometric parameters was published, reporting a strong statistical correlation up to 32 weeks of pregnancy and demonstrating that WJ is one of the major components of the umbilical cord in the second and third trimesters of pregnancy [11].

Other studies have also shown a strong correlation between the anthropometric parameters used to estimate gestational age and fetal weight with the area of WJ at ultrasonography [9,10,12-14]. Therefore, the objective of this study was to calculate a reference curve of the area of WJ in a cross-section of the umbilical cord as a function of gestational age in a population of low-risk pregnant women and to correlate these values with fetal weight, as calculated by routine ultrasonography.

## Materials and methods

This prospective, cross-sectional study was carried out between June 2005 and December 2006 in a total of 2,189 low-risk pregnant women of gestational ages ranging from 13 to 40 weeks, who had been referred for routine ultrasonography at the ultrasonography department of the University of Campinas' maternity and at one private imaging clinic in Campinas, Brazil.

Inclusion criteria comprised of: a low-risk pregnancy with a single living fetus, gestational age previously established by the date of the last menstrual period when reliable or by ultrasonography carried out in the first trimester, intact membranes, and normal amniotic fluid index [15]. The concept undertaken was to consider standards of a normal fetal growth under optimal environmental conditions [16] and for that, the exclusion criteria comprised of: diabetes, arterial hypertension of any etiology, fetal malformation, oligoamnios or polyhydramnios, clinical signs of intrauterine growth restriction or fetal macrosomia (symphisis-fundus height below or above, respectively, the lower or upper limits for gestational age), and morphological abnormalities in the umbilical cord or its blood flow (abnormal Doppler velocimetry).

A Toshiba-Power Vision 6000 ultrasonographic scanner, model SSA-370^® ^and a Voluson 730 PRO^® ^scanner with 3.5 mHz transabdominal convex transducers, adopted as standard for obstetric scans, were used for the ultrasonographic examinations carried out in this study. The pregnant women were submitted to routine ultrasonography in a semi-seated position during which biparietal diameter, head and abdominal circumferences and femur length were measured and estimated fetal weight (EFW) calculated according to Hadlock's formula [17]; in addition, other parameters routinely evaluated during pregnancy were also measured. Women who fulfilled all the inclusion criteria were informed of the nature of the study and invited to participate. Those who agreed to participate signed an informed consent form drawn up in accordance with the regulations of the Institutional Review Board, which approved the study protocol prior to commencement.

**Figure 1:**
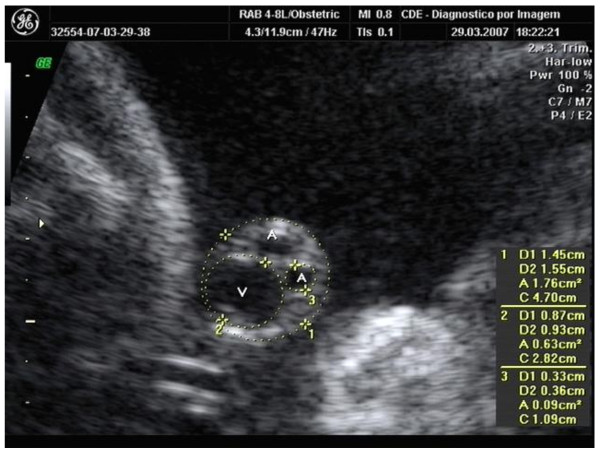
**Ultrasonographic measurement of the cross-sectional area of the umbilical cord (C), of the diameter of the umbilical vein (V) and of the umbilical artery (A)**. The area of Wharton Jelly (WJ) is WJ = C-V-2A.

The area of the umbilical cord was measured in all patients, together with the diameters of its vessels (arteries and vein) in a cross-sectional plane of the cord adjacent to its insertion in the fetal abdominal wall, at a maximum distance of 2.0 cm from the insertion point, using the elliptical calibrators of the ultrasound scanners at the outer edges of the cord and at the edges of the vessels in accordance with the method used by Raio *et al*. [10] and Weissman *et al*. [12] (Figure 1). The surface area of WJ was calculated according to the cross-sectional area of the umbilical cord from which the areas of the two arteries and the umbilical vein were subtracted (WJ = C-V-2A). The inter- and intra-observer variability of the measurements used to calculate the area of WJ were evaluated in a sub-sample of this population of women and were considered adequate [18].

For statistical analysis, first, the mean, standard deviation and median of the area of WJ in the umbilical cord were calculated in accordance with demographic and obstetrical characteristics, and statistical differences between them evaluated using the Kruskal-Wallis or Mann-Whitney non-parametrical tests. Next, the smoothed values of the 10^th^, 50^th ^and 90^th ^percentiles of these measurements were calculated for each gestational age, using third degree polynomial regression analysis, and resulting in the respective regression equations and coefficients of determination of the regression adjustment model (R^2^). Finally, the values of the area of WJ were correlated with the estimated fetal weight, and the linear coefficient of correlation (R) between them was calculated for two groups of cases: those up to 26 weeks of gestational age and those at more than 26 weeks. P values < 0.05 were considered significant.

## Results

The main characteristics of the 2,189 pregnant women are shown in Table 1. The majority was white, under 29 years of age and nullipara. There was no statistically significant difference in the area of WJ as a function of these characteristics; the only difference being with respect to gestational age.

**Table 1:** Means and medians of the area of Wharton's Jelly of the umbilical cord (mm^2^) in low-risk pregnancies, according to some demographic and obstetric characteristics

Characteristics	n^£^	Mean	SD	Median	p-value
**Ethnicity**					0.7011*
White	1756	110.7	56	106.9	
Non-white	433	109.3	56	107.3	
**Age (years)**					0.5204*
≤ 29	1254	109.9	56.9	106.4	
> 29	935	111.1	54.8	107.4	
**Parity**					0.0342*
Nullipara	1168	107.7	55.5	106.1	
≥ 1	1021	113.4	56.4	107.5	
**Gestational age (US)**					< 0.0001**
13	18	18.3	6.9	18.7	
14	43	23.3	7.5	23.4	
15	59	27.2	8.1	26.5	
16	60	39.5	13.5	35.8	
17	61	44.1	17.0	41.7	
18	60	45.3	18.1	41.4	
19	62	69.3	21.3	66.2	
20	110	67.9	22.1	64.4	
21	102	81.6	32.1	76.6	
22	91	86.7	33.2	80.7	
23	82	95.5	35.1	87.1	
24	60	115.2	35.0	115.6	
25	59	113.5	37.6	114.3	
26	62	120.2	37.2	120.9	
27	62	125.6	36.8	128.9	
28	92	137.0	51.3	136.9	
29	80	137.3	48.4	133.6	
30	91	141.1	49.8	125.3	
31	103	142.0	50.9	137.4	
32	101	142.0	51.3	132.7	
33	95	135.2	56.5	126.8	
34	102	137.9	44.0	136.0	
35	121	141.5	49.4	136.1	
36	102	135.6	45.7	130.4	
37	99	134.2	48.4	129.0	
38	94	134.7	50.7	121.7	
39	59	137.6	44.6	144.8	
40	59	151.9	52.0	143.3	

^£ ^N = 2189 * Mann-Whitney test ** Kruskal-Wallis test US: ultrasound

**Figure 2:**
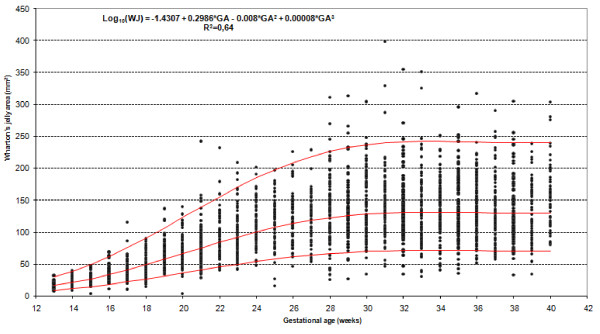
**Relationship between the area of Wharton's Jelly of the umbilical cord and gestational age**. The lines correspond to the 10^th^, 50^th ^and 90^th ^percentiles.

Table 2 shows the estimated 10^th^, 50^th ^and 90^th ^percentiles of the area of WJ for each gestational age between 13 and 40 complete weeks. To calculate the regression equation that defines the area of WJ according to gestational age (GA), the following was obtained: Log_10_(WJ) = -1.4307 + 0.2986*GA - 0.008*GA^2 ^+ 0.00008*GA^3^, for which the degree of adjustment (R^2^) was 0.64. Figure 2 shows the curve of these percentile values of the area of WJ according to gestational age. Note that values increase linearly until around 32 weeks when they reach a plateau, tending to stabilize from then onwards.

**Table 2:** Estimated values of percentiles of the area of Wharton's Jelly (mm^2^), according to gestational age

GA-US	p10	p50	p90	n
13	8.81	16.30	30.15	18
14	11.52	21.27	39.30	43
15	14.70	27.13	50.08	59
16	18.34	33.84	62.43	60
17	22.39	41.31	76.19	61
18	26.79	49.41	91.14	60
19	31.44	57.99	106.95	62
20	36.23	66.83	123.25	110
21	41.05	75.71	139.63	102
22	45.77	84.41	155.68	91
23	50.27	92.72	171.00	82
24	54.46	100.44	185.23	60
25	58.24	107.41	198.09	59
26	61.56	113.53	209.37	62
27	64.38	118.73	218.95	62
28	66.68	122.97	226.77	92
29	68.48	126.28	232.88	80
30	69.79	128.71	237.36	91
31	70.68	130.35	240.39	103
32	71.20	131.30	242.15	101
33	71.41	131.69	242.87	95
34	71.39	131.66	242.80	102
35	71.22	131.34	242.21	121
36	70.97	130.88	241.36	102
37	70.71	130.41	240.52	99
38	70.51	130.08	239.97	94
39	70.45	130.02	239.97	59
40	70.58	130.37	240.83	59

Equation: Log_10_(WJ) = -1.4307 + 0.2986*GA - 0.008*GA^2 ^+ 0.00008*GA^3^

GA-US: gestational age according to ultrasonography.

**Figure 3:**
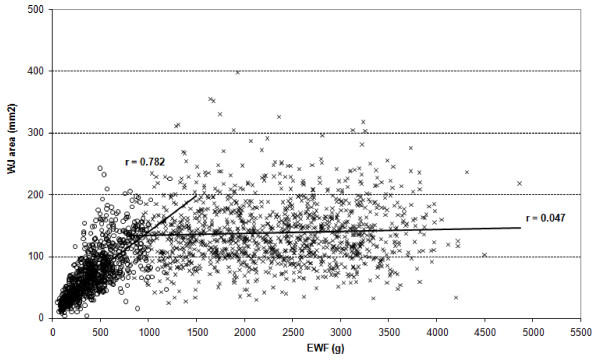
**Correlation between the area of Wharton's Jelly and estimated fetal weight (EFW) for low-risk pregnancies up to 26 weeks (r = 0.782) and above 26 weeks (r = 0.047)**.

Figure 3 shows the correlation between the measurement of the area of WJ and fetal weight as estimated by ultrasonography. This correlation increases linearly until 26 weeks of gestational age (R = 0.782), remaining practically constant from this gestational age onwards (R = 0.047).

## Discussion

This study shows a direct relationship between gestational age and the area of WJ in the umbilical cord. There is an increase in the area of WJ as a function of gestational age until around 32 weeks, after which these measurements remain practically stable until the end of pregnancy. Our findings also show a positive and linear relationship between estimated fetal weight and the area of WJ, but only until the 26^th ^week of gestational age, since from then onwards there is almost no change in the area of WJ compared to estimated fetal weight.

Previous case reports have shown a correlation between the presence of thin cords or a reduced area of WJ and fetal loss, premature births and inadequate fetal growth [7]. In 1967, studies were initiated on the macro and microscopic structure of the umbilical cord. Later, other investigators became interested in studying the tissue components of the umbilical cord. In 1983, Klein & Meyer [2] showed the macromolecular diffusion in WJ in relation to hyaluronic acid, one of its principal components. In 1994, Weissman *et al*. [12] presented a reference curve for the diameter of the umbilical cord and its vessels, which had not existed in the literature up to that time. Using the values of the diameters of the umbilical cord and its vessels, these investigators calculated the area of WJ at the different gestational ages and reported a peak at around 34 weeks.

Raio *et al*. [10] described a reference curve for the cross-sectional area of the umbilical cord and its vessels using a slightly different technique in which they viewed the cord through a cross-section, the same technique used in the present study. These investigators found a correlation between the cross-sectional area of the umbilical cord and fetal anthropometric parameters. Using this same technique, Ghezzi *et al*. in 2001 [11] established a curve of the area of WJ in a total of 659 low-risk pregnancies of 15-41 weeks. In fact, in 1994, Weissman *et al*. [12] had already defined normal values of the estimated area of WJ in 368 uncomplicated pregnancies, and reported differences in the values obtained, which varied between 13 and 27% depending on gestational age. One possible explanation for these results may be the different techniques used for measuring the umbilical cord. This same study reported that the ratio of the area of WJ in relation to the total area of the umbilical cord decreased significantly as gestational age increased, probably because of a reduction in the amount of water, one of its principal components.

In fact, WJ is the major component of the umbilical cord in the second and third trimesters [4]; therefore, if the area of the umbilical cord reaches its peak at around 31-32 weeks [19], the area of WJ would be expected to follow the same pattern.

However, the results of the present study are closer to those reported by Ghezzi *et al*. [11] and by Togni *et al*. [13,14], although values are slightly higher. These investigators studied 312 pregnant women of 24-39 weeks of pregnancy and described reference curves of the cross-sectional areas of the umbilical cord and its components, as well as the area of WJ, also reporting an increase at around 32 weeks followed by a plateau at around 35 weeks with values decreasing from 36 weeks onwards.

The correlation between the area of WJ and anthropometric parameters (which are used to calculate fetal weight) is generally weak. Togni et al. [13], for example, reported a correlation of only 0.240 between the area of WJ and estimated fetal weight. However, Ghezzi *et al*. [11] suspected this weak correlation to be a result of the overlap of two different situations as a function of gestational age; i.e., a strong correlation for earlier gestational ages and a weak correlation for later gestational ages. These results were exactly the ones found in the present study. On the other hand, a recent study did not find any correlation between the WJ area and de Umbilical Coiling Index during pregnancy [20].

Investigators have attempted to propose reference curves for the area of the umbilical cord and its components since 1994, and have carried out evaluations on the area of WJ in the umbilical cord. A possible strength of the present study is that it has the largest sample size described up to the present time and the results obtained are in agreement with previously obtained values. Our parameters should serve as a reference, mainly in cases in which diseases such as diabetes, arterial hypertension, and intrauterine growth restriction are suspected that may interfere with fetal development, and in which there may be changes in the morphology and in the function of the umbilical cord and in the area of WJ. For the specific purpose of using these measurements as possible predictors for fetuses classified as large for gestational age or small for gestational age, their performance was poor [21]. Nevertheless, appropriate validation of these curves is necessary to confirm the usefulness of these parameters. This represents a challenge for future research studies.

## Abbreviations

A: area of the umbilical arteries; C: area of the umbilical cord; EFW: estimated fetal weight; GA: gestational age; V: area of the umbilical vein; WJ: Wharton Jelly.

## Competing interests

The authors declare that they have no competing interests.

## Authors' contributions

CB and JGC had the original idea for the study and wrote the research protocol. CB and EFM were responsible for implementing exams and data collection. CB, JGC, MLC and FGS were responsible for planning the analytical approach and JVC performed the statistical analysis. CB wrote de first version of the manuscript. All of them gave inputs for interpretation of the results and in the manuscript, read and approved this final version of the manuscript.
